# Low APACHE II and ASA score predicts survival in patients with perforated peptic ulcer

**DOI:** 10.1186/1757-7241-20-S2-P29

**Published:** 2012-04-16

**Authors:** David Levarett Buck, Morten Vester-Andersen, Morten Hylander Møller

**Affiliations:** 1 The Emergency Department, Holbæk Hospital, Denmark; 2 Department of Anaesthesiology and Intensive Care Medicine, Copenhagen University Hospital Herlev, Denmark; 3 Department of Anaesthesiology and Intensive Care Medicine, Copenhagen University Hospital Bispebjerg, Denmark

## Background

Mortality and morbidity following perforated peptic ulcer (PPU) is substantial with mortality proportions up to 25-30%. The limited number of beds at the intensive care units emphasizes the importance of individual risk stratification. Accurate and early identification of high-risk and low-risk patients is necessary to plan and target the level of peri- and postoperative monitoring and treatment. At present, clinical prediction rules are not routinely used in PPU patients. The aim of the present study was to compare the performance of four clinical prediction rules in PPU: the Boey score, the APACHE II score, the ASA score, and the sepsis score.

## Methods

Patients surgically treated for PPU between January 1st, 2008 and December 31st, 2009 at seven gastrointestinal departments in Denmark was consecutively included. Pregnant and breastfeeding women, non-surgically treated patients, patients with malignant ulcers, and patients with perforation of other organs were excluded. The present study was approved by The Danish Data Protection Agency.

The primary outcome measure was 30-day mortality rate. The clinical prediction rules’ ability to distinguish survivors from non-survivors was evaluated by the area under the receiver operating characteristic curve (AUC), the positive predictive values (PPVs), i.e. the risk of dying within 30-days of surgery given a score above a certain threshold, and the negative predictive values (NPVs), i.e. the risk of surviving given a score below a certain threshold.

## Results

117 patients were included. Median age was 70 years (25-92 years), 51 % of the patients were females, and 73 % of the patients had at least one co-existing disease. The 30-day mortality proportion was 17 % (20/117). The AUCs: the Boey score, 63 %; the sepsis score, 69 %; the ASA score, 73 %; and the APACHE II score, 76 %. Overall, the PPVs of all four prediction rules were low (24-29 %), and the NPVs high (87-97 %).

## Conclusion

Especially APACHE II score (NPV 97 %), and ASA (NPV 93 %) score predicts good outcome in PPU patients with a high degree of precision. The sepsis score (NPV 90 %), and Boey score (NPV 87 %) perform worse. However, all clinical scores predict mortality poorly in patients with PPU.

